# Dual-Fuel-Driven Bactericidal Micromotor

**DOI:** 10.1007/s40820-015-0071-3

**Published:** 2015-11-13

**Authors:** Ya Ge, Mei Liu, Limei Liu, Yunyu Sun, Hui Zhang, Bin Dong

**Affiliations:** Institute of Functional Nano & Soft Materials (FUNSOM), Jiangsu Key Laboratory for Carbon-Based Functional Materials & Devices and Collaborative Innovation Center (CIC) of Suzhou Nano Science and Technology, Soochow University, Suzhou, 215123 Jiangsu People’s Republic of China

**Keywords:** Micromotor, Self-propelling, Bacterial killing, Janus, *E. coli*

## Abstract

In this paper, we report fabrication of the bimetallic Janus microsphere, a magnesium microsphere with a silver surface coating, through thermal evaporation technique. Because of the Janus structure, this micromotor can be propelled in two different directions by the surface silver or magnesium ‘engine’ and hydrogen peroxide or water fuel. In addition, due to the bactericidal property of silver, this autonomous micromotor is capable of killing bacteria in solution. As compared to the static one, the micromotor is able to kill the bacteria at a much faster rate (about nine times of that of the static one), demonstrating the superiority of the motion one. We thus believe that the micromotor shown in the current study is potentially attractive for the environmental hygiene applications.

**Electronic supplementary material:**

The online version of this article (doi:10.1007/s40820-015-0071-3) contains supplementary material, which is available to authorized users.

## Introduction

In the past decade, self-propelling nano- or micro-motors have attracted more and more attentions [1–6] due to their unique properties compared to the other nano/micro devices, such as their capability of achieving controlled cargo capture [7], transportation [8], release [9], and the more efficient delivery or faster recognition kinetics [10] caused by their autonomous motion process, which hold great promise in a wide range of applications, including drug delivery for biomedical field [11], oil droplet removal [12] for environmental remediation or detoxification [13]. These tiny devices are able to harvest chemical energies from the surrounding environment and convert them into mechanical work to realize autonomous movement in a liquid environment. A variety of propulsion mechanisms have been developed in order to realize the self-propelling behavior of a micromotor, which include bubble recoil [14], diffusiophoresis [15], and interfacial tension-induced propulsion [16]. In addition, since there is normally no control over the directionality of a moving micromotor, external fields such as magnetic field [17] or ultrasound [18] are generally applied to manipulate its moving direction. Although a variety of fuels such as hydrogen peroxide [19], acid [14], bromine, and iodine [20] have been developed to propel the micromotor, most of them are toxic for use in biomedical application and may even cause the environmental problems. For the purpose of protecting the environment and realizing better biocompatibility, it is desirable to develop micromotors that are environmentally friendly.

Recently, magnesium-based micromotor propelled by the magnesium and water reaction have been developed, which is capable of moving autonomously in a number of aqueous solutions, such as simulated body fluid, low concentration sodium carbonate, and sodium chloride solution [21, 22]. Because of the environmentally friendly nature of the magnesium-based micromotor, it has been demonstrated that they have potentials in a variety of biomedical or environmental applications. For example, Guan et al. have shown that the Pt/Mg bimetallic micromotor exhibits the excellent hemolytic property, which is an indication of its good biocompatibility [21]. By capping part of the magnesium microsphere with a thermally responsive polymer, i.e., poly(*N*-isopropylacrylamide), a water-driven micromotor, which is capable of achieving controlled drug release upon heat treatment, has been developed [23]. Recently, by depositing a thin layer of gold or titanium dioxide onto the surface of the magnesium microsphere, Wang et al. have developed the autonomous micromotor (Au/Mg and TiO_2_/Mg), which is able to collect spilled oil droplet for water cleaning (in the case of Au/Mg) [22] or degrading chemical or biological warfare agents for environmental remediation purpose (in the case of TiO_2_/Mg) [10]. Despite these progresses, to the best of our knowledge, few studies have realized the bacteria killing utilizing the magnesium-based micromotor.

In this paper, we report the Ag/Mg bimetallic micromotor which exhibits bactericidal capability. The micromotor, which is obtained through thermal evaporation method, possesses a Janus feature and can be self-propelled in two directions based on hydrogen peroxide or water fuels. Since silver and its ions have inhibitory effects on the bacteria and can be utilized as bactericidal or antimicrobial agents, the resulting micromotor shows excellent anti-bacterial property for bacteria, such as *E. c* *oli*. In addition, as compared to the static one, the moving micromotor exhibits a much faster killing rate. This can be attributed to the more silver ions released from the motion one as opposed to the static one and the motion-based solution mixing process, which cause the dissolved silver ions to reach the bacteria in a shorter time. The amount of bacteria killed in the case of the micromotor is about ninefold of that in the case of the static one, demonstrating the superiority of the autonomous micromotor. The self-propelling and fast bacterial killing property shown in this study makes current micromotor an attractive candidate for the environmental hygiene applications.

## Experimental

### Materials

Magnesium microspheres with diameters of 20–30 μm are obtained from Tangshan Weihao Magnesium Company. Silver, 99 % purity, is obtained from Zhong Nuo Advanced Material (Beijing) Technology Company. Sodium bicarbonate is purchased from Sigma-Aldrich Company. *E. c* *oli* is obtained from J&K Chemical Company. LIVE/DEAD Baclight staining kit is obtained from Molecular Probes Incorporation.

### Fabrication of Ag/Mg Janus Microsphere

The Mg microsphere is first dispersed in ethanol solution with a concentration of 50 mg mL^−1^ under ultrasonication and then placed on a glass substrate. The substrate is then placed inside the vacuum chamber of a thermal evaporator (NANO36, Kurt J. Lesker Company), where 20-nm Ag is deposited as indicated by the quartz crystal microbalance inside the chamber. After the Ag deposition, the Mg microsphere with the Ag surface layer is released into solution through ultrasonication.

### Characterization

Scanning electron microscopy (SEM) images and energy-dispersive X-ray analysis (EDX) are obtained on a Carl Zeiss Supra 55 scanning electron microscope. For the autonomous movement study, the Ag/Mg micromotor is first placed in aqueous solution containing 1 M NaHCO_3_. The self-propelling behavior is then observed and captured by utilizing a Nikon Eclipse 80i optical microscope. The data analysis of the moving behavior of the micromotor is carried out by utilizing PhysVis software. For the catalytic micromotor, the Ag/Mg structure is placed in aqueous solution containing 3 % hydrogen peroxide. The released silver ion concentration in the solution is measured on a Varian AA240FS-GTA120 atomic absorption spectroscopy.

### Anti-bacterial Activity of the Micromotor

In a typical experiment, 1 mL *E*. *coli* (1 × 10^10^ CFU mL^−1^) incubation solution is first centrifuged at 10,000 rpm. After removing the supernatant, 5 mg Ag/Mg micromotors (corresponding to ~3.5 × 10^5^ in number) are then added to 200 μL *E*. *coli* suspension containing 1 M NaHCO_3_. After 10 min, the mixture is first centrifuged at 1000 rpm to remove the micromotor and then centrifuged again at 10,000 rpm to obtain the *E. c* *oli* bacteria. The isolated *E*. *coli* bacteria are then mixed with 6 μM Syto-9 or 30 μM propidium iodide solution following the procedure shown in the LIVE/DEAD Baclight bacterial viability kit. After staining in the dark for 15 min, the *E*. *coli* bacteria are centrifuged at 10,000 rpm and re-dispersed in water. For the live and dead bacteria counting, 2 μL of solutions containing the bacteria is drop cast on a cover glass, which is then subject to fluorescence microscopic analysis. The captured fluorescence images are analyzed by utilizing ImageJ software.

## Results and Discussion

**Fig. 1:**
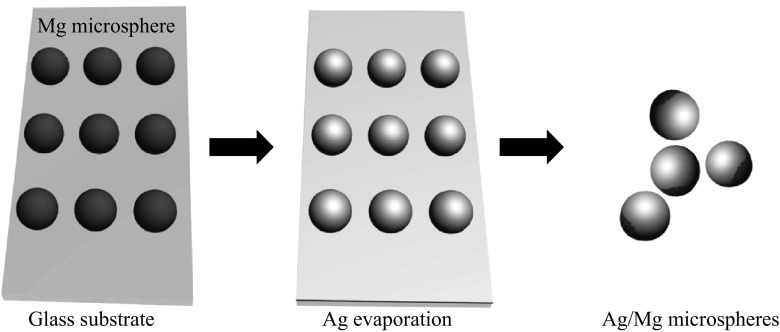
Schematic illustrates the fabrication process of the Ag/Mg bimetallic Janus microsphere

**Fig. 2:**
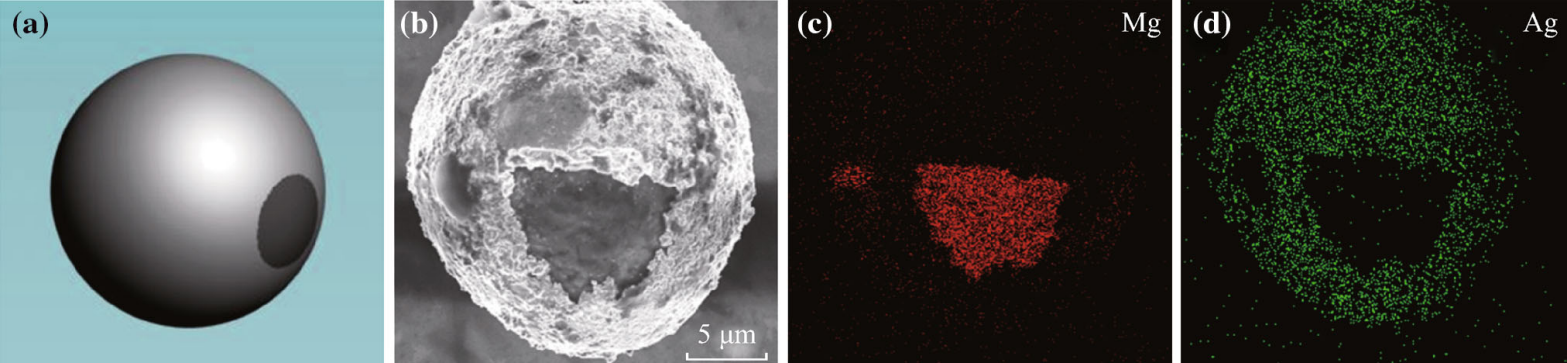
**a** Schematic image of the Ag/Mg microsphere. **b** Scanning electron microscopy image of Ag/Mg Janus structure. Energy-dispersive X-ray analysis of the structure shown in (**b**) for **c** Mg and **d** Ag, respectively

Figure 1 illustrates the fabrication process of the Ag/Mg micromotor based on the thermal evaporation method. Mg microparticles are first placed on a cover glass. After depositing 20-nm-thick silver onto the surface of the magnesium microspheres, Ag/Mg structures are formed, which are then removed from the cover glass to the aqueous solution through ultrasonication. SEM is used to confirm its morphology. Figure 2b shows a typical SEM image of the resulting Ag/Mg Janus microsphere, which has a Janus feature. The microparticle has a spherical shape with a diameter of approximately 25 μm. Part of its surface is covered with a thin layer of silver, whereas the rest is the exposed magnesium. EDX is used to confirm the composition of this structure. As can be seen from the elemental mapping of the resulting structure shown in Fig. 2c, d, the signal from Ag covers most of the surface of the microsphere, while the signal from Mg concentrates at the bottom part. The EDX analysis corresponding the elemental mapping and the corresponding weight percent of Ag and Mg are shown in the supporting information as Fig. S1 and Table S1, respectively. These results, together with the morphological structure shown in Fig. 2b, confirm the Ag/Mg microsphere structure with a Janus feature, i.e., the silver surface coating only decorates part of the surface of the magnesium microsphere.

**Fig. 3:**
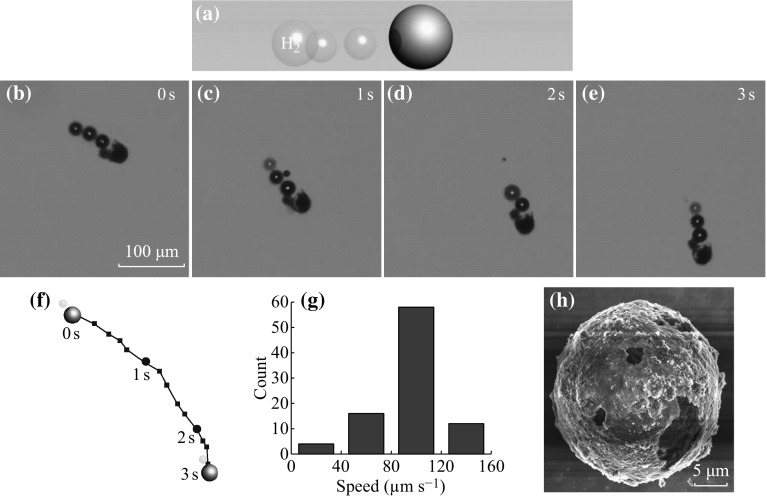
**a** The autonomous movement of the Ag/Mg micromotor in 1 M NaHCO_3_. **b–** **e** Time-lapse images taken at 1 s interval obtained from Video S1 in the supporting information indicate a typical motion of an Ag/Mg Janus micromotor in 1 M NaHCO_3_. **f** The corresponding moving trajectory of the micromotor shown in (**b–** **e**). **g** Histogram of the moving velocity of the Ag/Mg Janus micromotor. **h** SEM image showing the morphology of the residual silver layer obtained after the complete consumption of the magnesium microsphere

In order to realize the autonomous movement of a micromotor, the asymmetry is the foundation [24, 25]. Asymmetry relies on either structural or compositional differences. These differences may result in the generation of uni-directional forces when exposed to a suitable fuel, leading to the propulsion of the whole structure and the autonomous movement. Based on this principle, a variety of asymmetric structures have been developed. For example, Posner et al. have reported an Au/Pt micromotor based on polystyrene spheres, which can move in hydrogen peroxide aqueous solution [26]. Similarly, diverse asymmetric structures have been constructed, which include segmented nanorod with different compositions, such as gold, nickel, and platinum [27], microtubes consisting of different metal layers of iron and platinum [28, 29], or even complicated irregular L-shaped micromotors [30]. For current Ag/Mg micromotor, it possesses an asymmetric Janus structure, as illustrated in Fig. 2. The magnesium is stable in water due to the formation of the passivation layer of Mg(OH)_2_ as a result of the magnesium and water reaction. However, in the presence of the NaHCO_3_, it will react with the Mg(OH)_2_ passivation layer [21], so that the reaction between magnesium and water can continue and generate hydrogen bubbles for the propulsion of the whole structure, as shown in Fig. 3a. Figure 3b–e shows a series of optical images taken at 1-s interval indicating the self-propelling behavior of the Ag/Mg micromotor. It can be seen clearly that the bubbles are generated from the magnesium microsphere inside the silver shell, thus propelling the whole structure. The corresponding movie is shown as Video S1 in the supporting information. Based on this movie, we are able to obtain the moving trajectory of this micromotor, as indicated in Fig. 3f, which is close to a linear moving fashion. The curvature in the moving trajectory of the Ag/Mg micromotor can be ascribed to the slight deviation of the driving force from the mass center of the microsphere [23]. By analyzing the moving trajectory of this micromotor, the velocity can be estimated to be around 83 μm s^−1^. Furthermore, we have studied the average moving speed by measuring a number of micromotors. The average moving velocity of the Ag/Mg micromotor is around 90 μm s^−1^, as shown in the histogram (Fig. 3g). With the continuous autonomous movement and the consumption of the magnesium material, the Ag/Mg micromotor will finally stop. The total moving time for the micromotor is approximately 15 min. The total moving distance of this micromotor is estimated to be around 8.1 cm. In addition, we have examined the morphology of the resulting structure of the micromotor after the magnesium is consumed. As can be seen from Fig. 3h, the remaining structure of the micromotor is a thin shell of silver, which also confirms the Janus feature of the Ag/Mg micromotor. Note that the mechanism of the water-driven Ag/Mg micromotor is based on the chemical reaction between magnesium and water, while the Au/Pt micromotor reported previously is based on electrocatalytic reaction between Pt and the hydrogen peroxide [26]. Since hydrogen peroxide fuel is toxic, the water-driven Ag/Mg micromotor is advantageous for the biological applications.

**Fig. 4:**
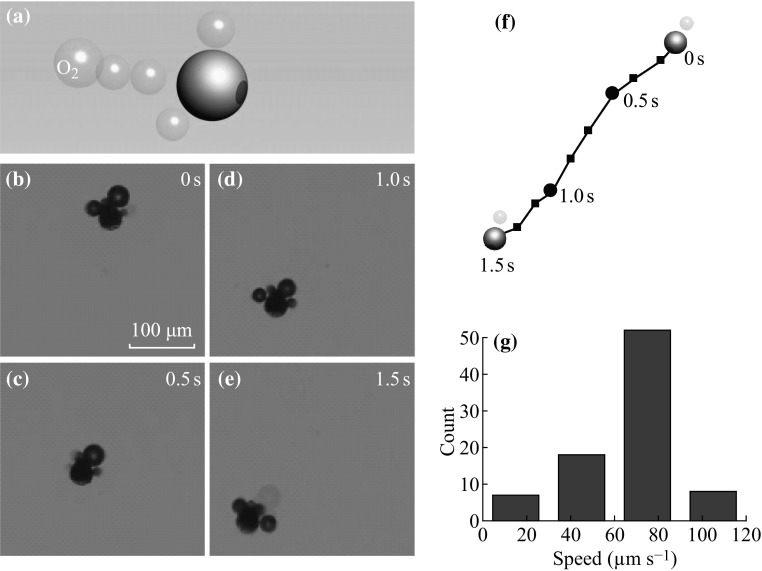
**a** The propulsion of the Ag/Mg micromotor in hydrogen peroxide. **b–** **e** Time-lapse images taken at 0.5 s interval from Video S3 in the supporting information show the movement of a typical Ag/Mg Janus micromotor in hydrogen peroxide solution. **f** The motion trajectory of this micromotor shown in (**b–** **e**). **g** The histogram showing the velocity distribution

As described previously, there are many applications related to the micromotor, which may require the propulsion of the micromotor under different environment. Therefore, the micromotor that can be driven by multiple fuels is desirable, which enable it to be utilized for various applications. For example, Wang et al. have reported the Al/Pd spherical hybrid micromotor propelled by three different fuels, i.e., acid, base, and hydrogen peroxide, which have potentials in diverse applications under different chemical environment [24]. One interesting aspect of the micromotor driven by multiple fuels is that it can move in different directions which are controlled by the location of the ‘engine’ part. In our current case, in addition to the magnesium and water reaction, silver is capable of catalyzing the decomposition reaction of the hydrogen peroxide to water and oxygen bubbles. It is thus possible to realize the autonomous movement of the micromotor in the presence of the hydrogen peroxide fuel, as illustrated in Fig. 4a. Figure 4b–e and the corresponding Video S2 in the supporting information show the self-propelling behavior of this Ag/Mg micromotor when placed in the hydrogen peroxide aqueous solution. Since the oxygen bubble is generated on the side containing silver surface coating in this case, the moving direction of this micromotor is toward the magnesium side of the Ag/Mg Janus microsphere (Fig. 4a–e), which is opposite to that of the water-driven micromotor. As can be seen from the moving trajectory of the micromotor shown in Fig. 4f, the motion behavior of this micromotor is almost a linear fashion, indicating the driving force to propel the micromotor deviates little from the mass center of the micromotor. In addition, based on the trajectory, the moving velocity of this micromotor can be estimated to be around 70 μm s^−1^. Furthermore, we have studied the average moving velocity of a number of micromotors. As can be seen from the velocity distribution histogram shown in Fig. 4g, the average speed is approximately 67 μm s^−1^. In addition, we have compared the moving speeds of the micromotor driven by magnesium and silver, which are 90 and 67 μm s^−1^, respectively. However, the weight of the magnesium and silver is about 1.4 × 10^−5^ and 6.9 × 10^−8^ mg, respectively. Since the magnesium is much heavier than the silver surface layer, therefore, it means the silver can carry a weight which is 200 times of its body weight at a high speed of 67 μm s^−1^, indicating the catalytic reaction is more efficient to propel the micromotor. For the micromotor based on catalytic reactions, the micromotor will also stop moving after the fuel exhausts. In our current case, the total propulsion time in hydrogen peroxide is around 30 min, which is longer than that based on water-driven mechanism. The total distance that this Ag/Mg micromotor can travel based on catalytic mechanism is around 12 cm, which is around 1.5-fold of that of water-driven one.

**Fig. 5:**
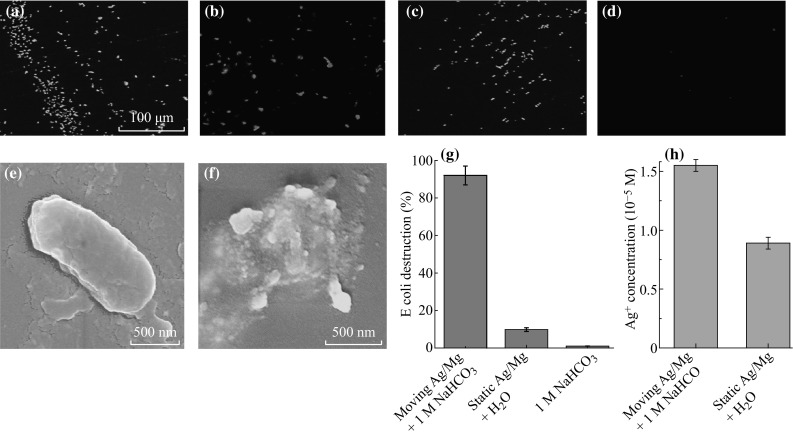
Fluorescence microscopic images of the stained live (**a**) and dead (**b**) *E. coli* bacteria after adding the moving Ag/Mg bimetallic micromotor for 10 min. Fluorescence images of the live (**c**) and dead (**d**) bacteria after treating with the same amount of static Ag/Mg microsphere for 10 min. SEM images indicate the morphology of the live (**e**) and dead (**f**) *E. coli* bacteria. **g** Percentage of the dead *E. coli* bacteria for the motion and static Ag/Mg microsphere. **h** The concentration of Ag^+^ released from the motion and static Ag/Mg microsphere

Since the micromotor synthesized in the current study can move autonomously in the salt solution with a layer of silver on its surface, which is known to be an excellent material for its bactericidal property for a wide spectrum of bacteria [31], we thus further studied its bacterial killing behavior [32]. We adopt the *E*. *coli* as the model bacteria, which is a threat to the human health [33]. In order to examine the effect of the micromotor on the bacteria, we first add the Ag/Mg micromotor to the solution containing *E*. *coli*. Live/dead staining method is then utilized to determine the viability of the bacteria. Live and dead bacteria can be separately stained with Syto-9 and propidium iodine, which can be easily distinguished under the fluorescence microscope by their corresponding green and red fluorescence emission, respectively. The percentage of the killed bacteria is then estimated by analyzing the captured fluorescence images using ImageJ software. Base on this method, we first evaluate the bacterial killing effect of the Ag/Mg micromotor. After exposing the bacterial solution to the Ag/Mg micromotor for 10 min, we examine the live and dead bacteria, respectively. As can be seen from the fluorescence image shown in Fig. 5a, the live bacteria have an elliptical shape and can be stained by the green Syto-9 dye, which is basically the stain of the bacterial membrane due to the integral membrane of the live bacteria. Furthermore, we have examined the morphology of the intact *E*. *coli* bacteria using SEM. As can be seen from Fig. 5e, the *E*. *coli* bacterium has a regular shape and a smooth surface morphology, indicating its intact structure. On the contrary, because of the broken bacterial membrane of the dead bacteria killed by the silver ions released from the silver layer of the Ag/Mg bimetallic micromotor, the nuclei can be stained with the red fluorescent propidium iodine dye, which accounts for the red fluorescence emission of the dead bacteria shown in Fig. 5b. The SEM examination further confirmed the ruptured structure of the dead E-coli bacteria, as indicated in Fig. 5f. The percentage of the dead bacteria is estimated in this case to be around 90 % (Fig. 5g). Note that the NaHCO_3_ solution has negligible influence on the *E*. *coli* viability (Fig. 5g). Furthermore, we have performed a control experiment by adding the same concentration of Ag/Mg bimetallic structures directly to the solution containing *E*. *coli* bacteria in the absence of the NaHCO_3_, which remains static inside the solution in this case. After 10 min, the static Ag/Mg bimetallic structure can still kill the *E*. *coli* bacteria, as illustrated in Fig. 5c, d. The killing efficiency is estimated to be around 10 % (Fig. 5g), which is much lower than that of its moving counterpart. In order to explain this phenomenon, we have carried out the atomic absorption spectroscopy to study the released silver ion concentration in the solution. For the motion and the static Ag/Mg micromotors, the silver concentrations are measured to be around 1.55 × 10^−5^ and 0.89 × 10^−5^ mol L^−1^ (Fig. 5h), respectively, i.e., the moving micromotor releases more silver ions than the static one. This result is consistent with that reported in the literature [34], where more metallic ions are released from the micromotor due to the corrosion. We thus attributed the enhanced bacterial killing capability in the case of the micromotor to the higher silver ion concentration and the motion-induced solution mixing process, which enables the silver ion to be delivered more quickly to the *E. c* *oli* compared to the static one.

**Fig. 6:**
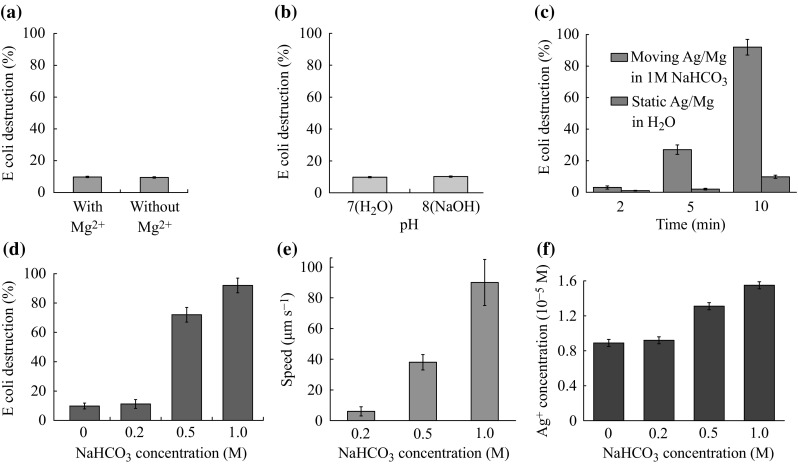
The influence of **a** the Mg ions and **b** the solution pH on the bacteria killing rate. **c** Time-dependent bacteria destruction. The effect of the NaHCO_3_ concentration on **d** the bacteria killing efficiency, **e** the micromotor speed and **f** the released silver ion concentration

In order to further confirm the different bacterial killing rates for motion and static micromotor, we have performed more control experiments. First, we have studied the effect of released Mg^2+^ on the bacterial killing behavior in the presence of NaHCO_3_. We have prepared two samples: one is the Ag/Mg Janus particle after dissolving the magnesium in 1 M NaHCO_3_; the other is a similar sample after removing the dissolved Mg ions by centrifugation and re-dispersing in 1 M NaHCO_3_. As can be seen from Fig. 6a, these two samples show similar bacteria killing efficiency, indicating Mg ions have negligible influence on the results. Second, since 1 M NaHCO_3_ solution has a pH of 8, we have further explored whether the pH will influence the bacterial killing behavior. We adjust the pH of the static Ag/Mg to 8 by adding NaOH (Note that Mg will not react with NaOH) and study the bacteria killing rate. As can be seen from Fig. 6b, the killing efficiency of static Ag/Mg micromotor at pH 7 and 8 has negligible difference. Third, we have performed the time-dependent *E*. *coli* killing experiment. As shown in Fig. 6c, the *E*. *coli* destruction ratio increases with time. Fourth, we have studied the influence of the NaHCO_3_ concentration on the bacteria killing rate. As shown in Fig. 6d, the bacteria killing efficiency is dependent on the NaHCO_3_ concentration. Higher NaHCO_3_ concentration leads to the higher bacteria killing percentage. In order to explain this phenomenon, we have investigated the moving velocity and the released silver ions at different NaHCO_3_ concentrations. As illustrated in Fig. 6e, f, both the micromotor speed and the amount of the released silver ions are dependent on the NaHCO_3_ concentration. Therefore, we can attribute the increased bacterial killing efficiency at higher NaHCO_3_ concentration to the higher moving velocity and more released silver ions.

## Conclusions

In conclusion, we fabricate a Janus micromotor consisting of magnesium microsphere with asymmetric surface silver coating by thermal evaporation method. Not only can this micromotor be self-propelled by two different fuels, they are also capable of killing bacteria due to the bactericidal property originating from the silver material. Because of the solution mixing process induced by the continuous motion and the more released silver ion, the self-propelled micromotor exhibits more efficient anti-bacterial property than the static one. The features shown in the current study, such as the easy fabrication, water driven, and excellent anti-bacterial behavior, make current micromotor potentially attractive for environmental hygiene applications.

## Electronic supplementary material

Below is the link to the electronic supplementary material.

Supplementary material 1 (wmv 151 kb)

Supplementary material 2 (docx 120 kb)

Supplementary material 3 (wmv 260 kb)
