# Supplementary material for: Dual-Fuel-Driven Bactericidal Micromotor
Source: Nanomicro Lett. 2015 Nov 13;8(2):157–64. doi: 10.1007/s40820-015-0071-3 (PMC6223669; doi:10.1007/s40820-015-0071-3)
Supplement: Supplementary file 2 — Supplementary material 2 (docx 120 kb) [file 40820_2015_71_MOESM2_ESM.docx]

Supporting Information for

**Dual Fuel Driven Bactericidal Micromotor**

Ya Ge, Mei Liu, Limei Liu, Yunyu Sun, Hui Zhang, Bin Dong*

Institute of Functional Nano & Soft Materials (FUNSOM), Jiangsu Key Laboratory for Carbon-Based Functional Materials & Devices and Collaborative Innovation Center (CIC) of Suzhou Nano Science and Technology, Soochow University, Suzhou, Jiangsu 215123, People’s Republic of China

*Corresponding author. E-mail: bdong@suda.edu.cn

**Video S1** The autonomous motion of Ag/Mg micromotors in 1 M NaHCO_3_ solution

**Video S2** The autonomous motion of Ag/Mg micromotors in hydrogen peroxide aqueous solution

**Fig. S1** The EDX analysis corresponding the elemental mapping shown in Fig. 2 in the main text

**Table S1** The weight percent of the different elements obtained from Fig. S1

**Fig. S1**

**Table S1**
